# Elucidating the Crucial Role of Poly *N*-Acetylglucosamine from *Staphylococcus aureus* in Cellular Adhesion and Pathogenesis

**DOI:** 10.1371/journal.pone.0124216

**Published:** 2015-04-15

**Authors:** Mei Hui Lin, Jwu Ching Shu, Li Ping Lin, Kowit yu Chong, Ya Wen Cheng, Jia Fu Du, Shih-Tung Liu

**Affiliations:** 1 Department of Medical Biotechnology and Laboratory Science, College of Medicine, Chang-Gung University, Taoyuan, Taiwan, Republic of China; 2 Department of Microbiology and Immunology, College of Medicine, Chang Gung University, Taoyuan, Taiwan, Republic of China; 3 Department of Medical Research and Development, Chiayi Branch, Chang Gung Memorial Hospital, Chiayi, Taiwan, Republic of China; National Institutes of Health, UNITED STATES

## Abstract

*Staphylococcus aureus* is an important pathogen that forms biofilms on the surfaces of medical implants. Biofilm formation by *S*. *aureus* is associated with the production of poly *N*-acetylglucosamine (PNAG), also referred to as polysaccharide intercellular adhesin (PIA), which mediates bacterial adhesion, leading to the accumulation of bacteria on solid surfaces. This study shows that the ability of *S*. *aureus* SA113 to adhere to nasal epithelial cells is reduced after the deletion of the *ica* operon, which contains genes encoding PIA/PNAG synthesis. However, this ability is restored after a plasmid carrying the entire *ica* operon is transformed into the mutant strain, *S*. *aureus* SA113Δ*ica*, showing that the synthesis of PIA/PNAG is important for adhesion to epithelial cells. Additionally, *S*. *carnosus* TM300, which does not produce PIA/PNAG, forms a biofilm and adheres to epithelial cells after the bacteria are transformed with a PIA/PNAG-expressing plasmid, pTX*icaADBC*. The adhesion of *S*. *carnosus* TM300 to epithelial cells is also demonstrated by adding purified exopolysaccharide (EPS), which contains PIA/PNAG, to the bacteria. In addition, using a mouse model, we find that the abscess lesions and bacterial burden in lung tissues is higher in mice infected with *S*. *aureus* SA113 than in those infected with the mutant strain, *S*. *aureus* SA113Δ*ica*. The results indicate that PIA/PNAG promotes the adhesion of *S*. *aureus* to human nasal epithelial cells and lung infections in a mouse model. This study elucidates a mechanism that is important to the pathogenesis of *S*. *aureus* infections.

## Introduction

The nosocomial and community-associated pathogen *Staphylococcus aureus* causes various human diseases. This organism is present ubiquitously on the skin and in the nasal cavities of healthy individuals, explaining why bacterial contamination incurs during medical procedures and the surgical implantation of medical devices causes nosocomial *S*. *aureus* infections [1]. Additionally, *S*. *aureus* nasal colonization is a major source of auto-infection [2,3]. The ability to adhere and subsequently form biofilms on indwelling devices also contributes to the most important pathogenic factor of nosocomial *S*. *aureus* infections [4]. The formation of biofilms by *S*. *aureus* is closely associated with the synthesis of poly *N*-acetylglucosamine (PNAG) also referred to as the polysaccharide intercellular adhesin (PIA). PIA/PNAG is a β-1,6-linked *N*-acetylglucosamine homopolymer and synthesized by enzymes encoded by the *ica* operon which consists of four genes (*icaA*, *icaB*, *icaC*, and *icaD*) [5,6]. As the main surface component in biofilm structures, PIA/PNAG mediates intercellular adhesion, leading to the accumulation of bacterial cells [7].

Colonization of *S*. *aureus* is initiated by adhesion to host cells via interactions between a family of bacterial adhesins and receptors on the surfaces of host cells [8]. It has been well established that many invasive *S*. *aureus* strains express a large number of adhesins [9,10]. Cell wall-anchored adhesins, which are well-studied members of this family, are also referred to as microbial surface components recognizing adhesive matrix molecules (MSCRAMM). These molecules include fibronectin (Fn)-binding proteins (FnBPA/-B), fibrinogen (Fg)-binding proteins (clumping factor A and B, ClfA/-B), and collagen-binding protein (Cna) [9], which bind to the extracellular matrix (ECM) proteins of host tissues. Another class of staphylococcal adhesins is secreted and includes extracellular adherence protein (Eap) and extracellular matrix protein-binding protein (Emp). These proteins display broad binding abilities to several extracellular matrix and plasma proteins [10,11]. Staphylococci also produce non-proteinaceous adhesins, including wall teichoic acid (WTA), lipoteichoic acid (LTA) and exopolysaccharides, including PIA/PNAG [12,13].

An earlier study found that a strain of *S*. *epidermidis* that is defective in *icaB* produces non-deacetylated PIA polymers. Since deacetylation is responsible for the stable attachment of PIA to bacterial surfaces [14], the mutant forms less biofilm than the wild-type strain, and does not effectively colonize skin epithelial cells [14]. Mutants that are defective in synthesizing PIA/PNAG are also susceptible to antibacterial agents and killing by polymorphonuclear leukocyte (PMN) [14–16]. These results indicated that the PIA of *S*. *epidermidis* contributes to cellular adherence and immune evasion [14–16]. This study showed that PIA/PNAG is crucial for *S*. *aureus* to adhere to nasal epithelial cells and for the development of lung infections in a mouse model, suggesting that PIA/PNAG promotes cellular adhesion and contributes significantly to staphylococcal pathogenesis.

## Materials and Methods

### Bacterial strains, plasmids and culturing conditions

*S*. *aureus* SA113 (ATCC 35556) produces PIA/PNAG and biofilms [17]. *S*. *aureus* SA113Δ*ica* contains a deletion in the *ica* operon and does not produce PIA/PNAG [18]. *S*. *epidermidis* O-47 is a clinical isolate, which produces biofilms and PIA [19]. *S*. *epidermidis* O-47Δ*icaB* which carries an *icaB* mutation was kindly provided by Professor F. Götz from Tübingen University, Germany. *S*. *carnosus* TM300 (ATCC51365) [20,21] is a strain that does not produce PIA/PNAG and biofilms. *E*. *coli* DH5α [22] and a restriction-deficient strain of *S*. *aureus*, RN4220 [23], were used as hosts for cloning. Plasmid pTX*icaADBC* contains the *ica* operon and expresses PIA/PNAG after induction with 0.5% xylose [5]. In this study, the *ica* operon with its promoter was amplified by PCR, using primers IcaF (5’-CGGGGTACCAAAATTCCTCAGGCGTATTAG) and IcaR (5’-ACATGCATGCACCGCGTGTTTTTAACATAG) and the *S*. *aureus* SA113 chromosome as a template. The PCR product was then cut with *Kpn*I and *Sph*I, and inserted into the *Kpn*I-*Sph*I sites in a shuttle vector, pGHL6 [24] to generate pC*ica*. *S*. *aureus* strains were cultured in tryptic soy agar (TSA) or broth (Oxoid, Basingstoke, United Kingdom) containing 0.5% glucose (TSBg) or 0.5% xylose (TSBx). *E*. *coli* was cultivated in LB medium. Antibiotic-resistant colonies were selected on media that contained tetracycline (5 μg/ml), chloramphenicol (10 μg/ml), or ampicillin (100 μg/ml).

### Biofilm assay

An overnight culture of *S*. *aureus* was diluted 200-fold with TSBg, of which 200 μl was added to the wells of a 96-well polystyrene microtiter plate and incubated for 24 h at 37°C. The amount of biofilm formed in each well was determined by a safranin staining method described elsewhere [25]. Each experiment was performed at least three times, and the samples in each experiment were prepared in six wells.

### Extraction and quantification of exopolysaccharides (EPS)

EPS was isolated according to methods described elsewhere [15,26] but with modifications. *S*. *aureus* was cultured in 10 ml TSBg in a petri dish overnight. The cells were scraped from the petri dish. Following centrifugation, cell pellets were incubated in 3 ml 0.5 M EDTA (pH 8.0) per gram wet weight at 100°C for 10 min. After centrifugation at 16000 x *g* for 45 min, the supernatant was diluted 100-fold and incubated with proteinase K (2 mg/ml) for 2 h at 37°C. The crude extract of EPS was stored at -80°C.

EPS was purified by dialyzing the crude extracts against distilled water for 24 h followed by incubation with DNase I (0.5 mg/ml), RNase A (0.5 mg/ml), lysostaphin (0.5 mg/ml), lysozyme (0.5 mg/ml) and proteinase K (4 mg/ml) for 16 h at 37°C. Following centrifugation at 28000 x *g* for 30 min at 4°C, the supernatant was filtered using a 0.45-μm filter and subsequently concentrated approximately five-fold by lyophilization. Next, the concentration of purified EPS was determined using a method described elsewhere [27]. Briefly, 200 μl of sample was incubated with 150 μl of 6 N HCl in boiling water for 3 h. For neutralization, 100 μl of 10 N NaOH was added to the solution. Subsequently, 500 μl of a freshly prepared acetylacetone solution containing 1.5 ml acetylacetone and 50 ml of 1.25 M Na_2_CO_3_ was added and incubated in a water bath at 90°C for 60 min. After it was cooled, 2.5 ml of 95% ethanol and 500 μl Ehrlich reagent (Sigma-Aldrich) were added to the solution. After incubation at room temperature for 45 min, the concentration of purified EPS was determined at A_535_, according to a standard curve established using glucosamine. The crude extract of PIA/PNAG was blotted onto a polyvinylidene difluoride (PVDF) membrane (Millipore, Billerica, MA) using a 96-well dot-blot apparatus. Following blotting, the membrane was dried and soaked in a solution containing 3% bovine serum albumin and 0.05% Tween-20 in phosphate-buffered saline (PBS). The membrane was then incubated at room temperature for 1 h in a solution containing 0.8 mg/ml wheat germ agglutinin conjugated with biotin (WGA-biotin) (Sigma-Aldrich). After washing four times with PBS, the amount of PIA/PNAG was detected using horseradish peroxidase-conjugated streptavidin, followed by chemiluminescence detection (Pierce). Dispersin B [28], which degrades PIA/PNAG, was purchased from Kane Biotech, Inc. (Winnipeg, Canada).

### Cell culture conditions and adherence assay

RPMI 2650 (ATCC CCL 30), which is a human nasal septum carcinoma cell line, was cultured in Eagle’s minimal essential medium with Earle’s balanced salts (MEM/EBSS, GIBCO) supplemented with 10% fetal bovine serum (FBS). Cells were then seeded with 5x10^5^ cells per well in 24-well plates and incubated at 37°C for 24 h until they reached confluence. For the adherence assay, bacterial cells were dispersed by six 2 s pulses of sonication with 4 s intervals at 4°C with a Vibra cell sonicator (Sonics, Newtown, CT) at a 40% duty cycle output control setting before they were added to the RPMI 2650 cells at a multiplicity of infection (MOI) of 50. The plate was centrifuged at 100 x g for 5 min and then incubated for 1 h to allow for adhesion. After the cells were washed with PBS twice to remove non-adherent bacteria, they were detached from the plate with a 0.25% trypsin-EDTA solution. Next, the number of bacteria in the solution was determined by colony forming unit (CFU) enumeration by plating the bacteria on agar medium. The average number of bacteria that adhered to each cell was calculated. *S*. *carnosus* TM300, which does not adhere to human cells, was used as a control.

### Scanning electron microscopy (SEM)

RPMI 2650 cells were grown on polycarbonate discs, which were placed into the wells of a 24-well tissue culture plate. Bacteria at an MOI of 50 were then added to the wells and incubated for 1 h. Next, the discs were washed three times with PBS and prepared for SEM examination as described elsewhere [29]. Finally, the samples were observed under a Hitachi S-5000 scanning electron microscope at the Microscopy Core Laboratory of the Chang Gung Memorial Hospital.

### Mouse model of lung infections

The experimental procedures involving animals were approved by the Chang Gung University Animal Care Committee (approval no. CGU12-132). All mice were housed in groups of five under temperature-controlled conditions and provided food and water at the Animal Resource Center of the Chang Gung University. Six- to eight-week-old male C57BL/6 mice were anesthetized by isoflurane inhalation, and their lungs were inoculated with 30 μl bacterial suspension, which contained 10^9^ CFUs in PBS, by intratracheal injection according to a method described elsewhere [30]. After bacterial inoculation, the mice were monitored closely every 12 h until the end of experiments. The mice were sacrificed at three days after infection. The lung tissue was excised, washed twice with PBS and homogenized in 1 ml sterile PBS. The homogenized lung tissue was centrifuged at 500 x *g* for 5 min, and the supernatant was plated on TSA. The number of colonized bacteria was determined by counting the colonies on the TSA plates. For histological analysis, lung tissues were fixed in formalin, embedded in paraffin, thin-sectioned, stained with hematoxylin and eosin (H&E), and observed under a light microscope.

### Solid-phase extracellular matrix (ECM) binding assay

Wells in 96-well plates were coated with 10 μg/ml fibronectin (Fn) (Sigma-Aldrich), fibrinogen (Fg) (Sigma-Aldrich) and collagen (Cn) (Sigma-Aldrich). ECM proteins (100 μl) were added to each well and incubated overnight at 4°C. After incubation, the wells were washed with PBS three times; 100 μl 10 μg/ml BSA was then added to each well and incubated at 37°C for 1 hr. Subsequently, 100 μl of bacterial suspension (5x10^7^ CFU) was added to the wells. Following incubation at 37°C for 1 hr, the plate was washed with PBS three times to remove the non-adherent bacteria. Moreover, the adhered bacteria were fixed by incubating the plate at 65°C for 40 min and stained with 0.1% crystal violet at room temperature for 5 min. Furthermore, the stain was extracted using 10% acetic acid, and its absorbance was measured at 595 nm in a microtiter plate reader (SpectraMax 340; Molecular Devices).

### Statistical analysis

The significant differences between the different strains in the adherence assays were analyzed using the Student’s *t*-test. Statistical analysis of the lung infection data was performed using the non-parametric Mann-Whitney test. The data were analyzed using GraphPad Prism version 5.0 software (La Jolla, CA, USA).

## Results

### PIA/PNAG influences cellular adherence of *S*. *aureus* to nasal epithelial cells

**Fig 1:**
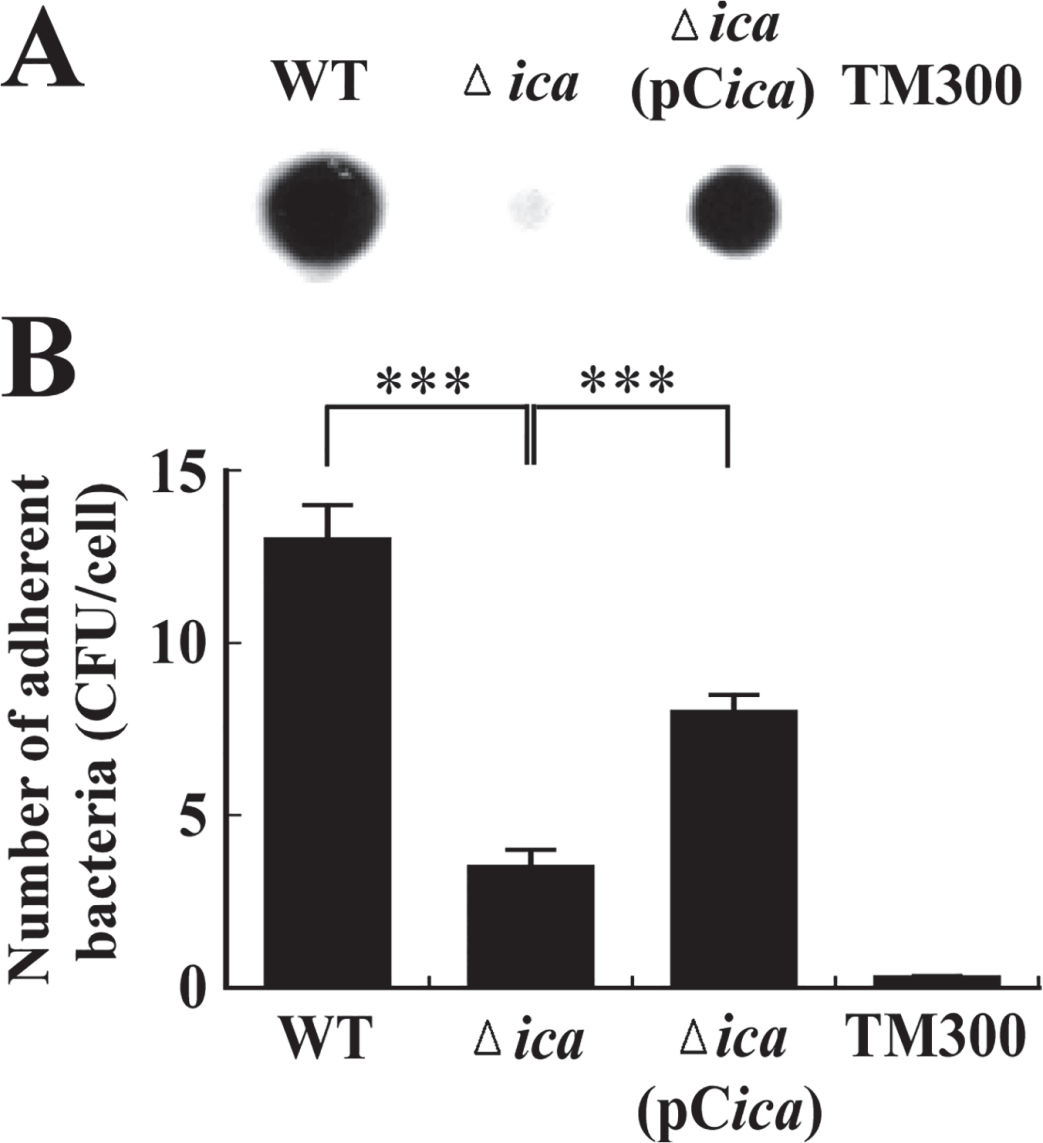
PIA/PNAG production and adherence of *S*. *aureus* to nasal epithelial cells. (A) PIA/PNAG was extracted from *S*. *aureus* strains and detected using WGA-biotin. Following incubation with HRP-streptavidin, PIA/PNAG was visualized by chemiluminescence detection. (B) The adherence of bacteria to RPMI 2650 cells was determined using an adherence assay. The number of bacteria that adhered to the cells was determined by CFU enumeration, and the average number of bacteria adhered to each RPMI 2650 cell was calculated. *S*. *carnosus* TM300 was used as a control. Significant differences are denoted with ***p-value < 0.001.

As is well known, PIA/PNAG has a major role in biofilm formation by *S*. *aureus*. This study examines how the expression of PIA/PNAG affects adherence of bacteria to the surface of RPMI 2650 nasal epithelial cells. Since *S*. *aureus* is intrinsically clumpy, the bacterial solution was sonicated to disperse the bacteria before inoculation to ensure their accurate enumeration. Furthermore, bacteria in culture plates were centrifuged at 100 x g for 5 min after they were added to the wells to ensure contact between the RPMI 2650 and the bacterial cells. Adhesion assay was performed after the growth of RPMI 2650 reached confluence to minimize potential enumeration errors that could arise from the attachment of bacteria to the surface of the dish. The results of the PIA/PNAG detection assay demonstrated that *S*. *aureus* SA113 produced a large amount of PIA/PNAG but *S*. *aureus* SA113Δ*ica* produced very little (Fig 1A). After *S*. *aureus* SA113Δ*ica* was transformed with pC*ica*, the synthesis of PIA/PNAG was partially restored (Fig 1A). Adherence assay revealed that the average number of *S*. *aureus* SA113 that adhered to each RPMI 2650 cell was 13 (Fig 1B). When the *ica* operon was deleted from strain SA113, i.e. SA113Δ*ica*, the adherent bacteria fell to 3.5 per cell (Fig 1B). This number increased to 8 when the mutant strain was transformed with pC*ica* (Fig 1B). Notably, the complementation of the mutant strain with pC*ica* did not result in the full recovery of adhesion to the RPMI 2650 cells, probably because the complementation strain did not produce as much PIA/PNAG as did the wild-type strain (Fig 1A).

**Fig 2:**
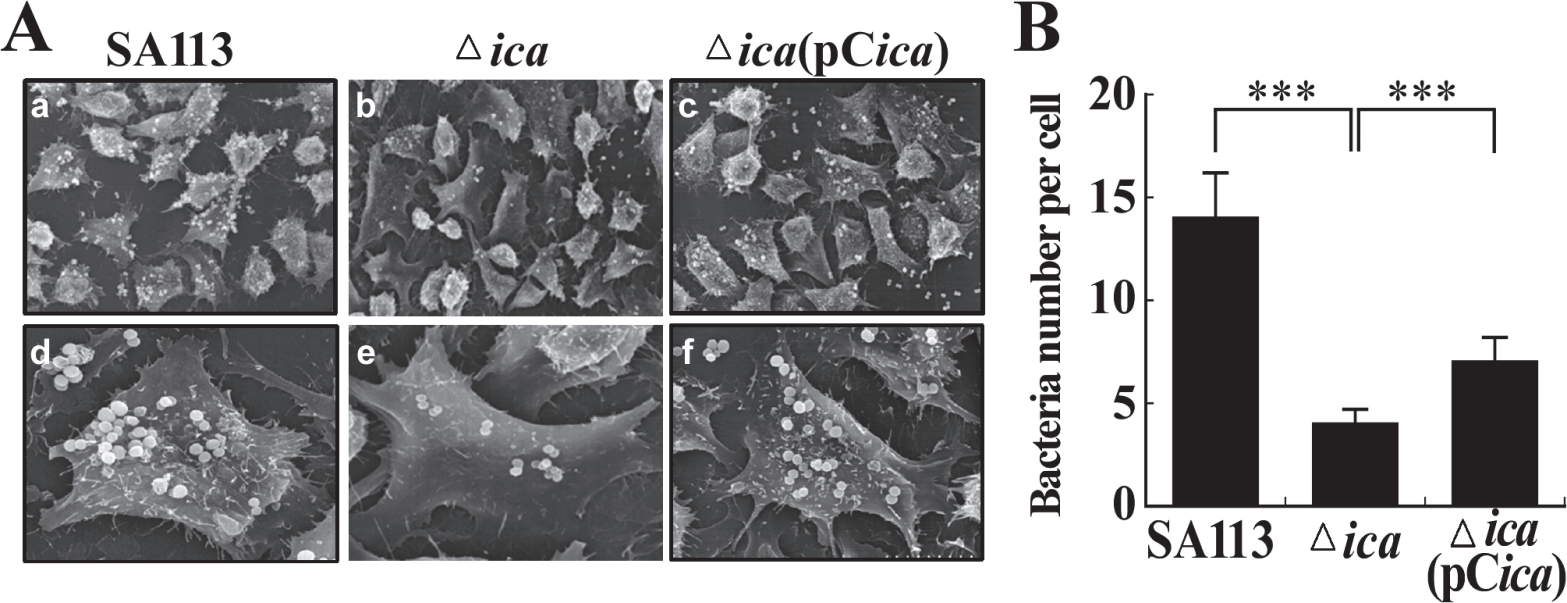
SEM images of adhesion of *S*. *aureus* to RPMI 2650 cells. (A) RPMI 2650 cells were incubated with *S*. *aureus* SA113 (a, d), SA113Δ*ica* (b, e) and SA113Δ*ica*(pC*ica*) (c, f). The images were captured at a magnification of 1000x (a, b, c) and 3000x (d, e, f). The number of bacteria that adhered to 400 cells was enumerated, and the average number of bacteria on each cell was calculated (B). Significant differences are denoted with ***p-value < 0.001.

A scanning electron microscopic (SEM) study also revealed similar results (Fig 2); enumeration of the bacteria on the surfaces of 400 RPMI 2650 cells revealed that the average numbers of adhered *S*. *aureus* SA113, SA113 Δ*ica*, and SA113 Δ*ica*(pC*ica*) per cell were 14, 4 and 7, respectively (Fig 2B), indicating that *S*. *aureus* does not adhere to epithelial cells efficiently without PIA/PNAG.

**Fig 3:**
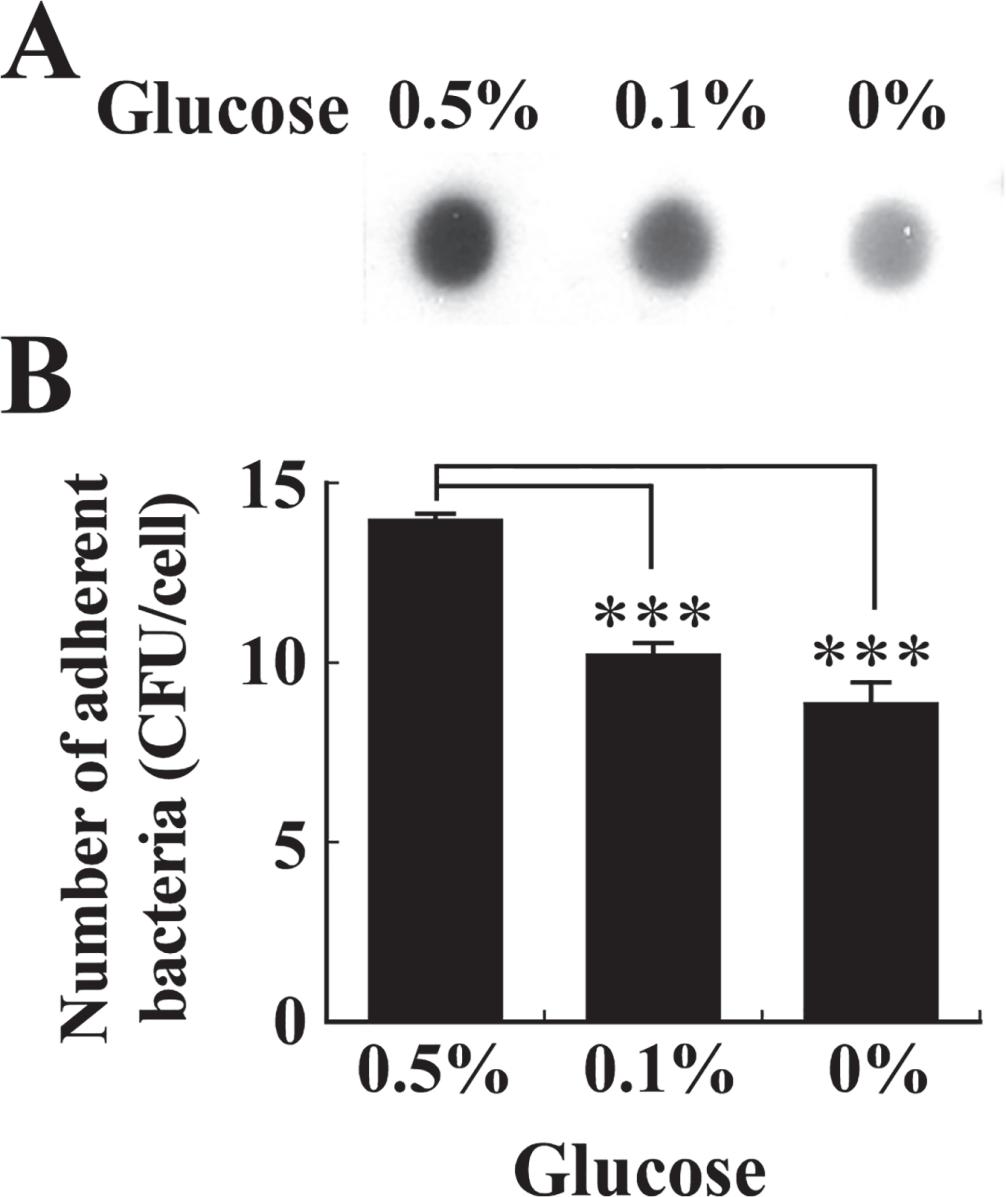
Effects of glucose on PIA/PNAG production and adherence of *S*. *aureus* to RPMI 2650 cells. *S*. *aureus* SA113 was cultured in glucose-containing TSB for 24 h. PIA/PNAG production (A) was determined using WGA-biotin. The average number of *S*. *aureus* SA113 adhered to each RPMI 2650 cell (B) was also determined using adherence assay. Significant differences are denoted with ***p-value < 0.001.

The amount of glucose in culture medium influences the expression of PIA/PNAG by *S*. *aureus* [31]. Therefore, in this study, the bacteria were cultured in media containing various concentrations of glucose. The results indicated that *S*. *aureus* SA113 produced more PIA/PNAG in TSB containing 0.5% glucose than in the medium containing 0.1% glucose or no glucose (Fig 3A). Additionally, PIA/PNAG production correlated with the ability of the bacteria to adhere to RPMI 2650 cells since the average number of the bacteria that were cultured in TSB with 0.5% glucose adhered to each cell was 14. The numbers dropped to 10 and 9 when the bacteria were cultured in TSB containing 0.1% glucose or no glucose, respectively (Fig 3B). Meanwhile, the glucose concentration in the medium did not appear to affect the ability of *S*. *aureus* SA113Δ*ica* to adhere to RPMI 2650 cells. These findings suggest that glucose promotes the production of PIA/PNAG, ultimately improving the adhesion of bacteria to epithelial cells.

### Enhancement of adherence of *S*. *carnosus* TM300 to RPMI 2650 cells by PIA/PNAG

**Fig 4:**
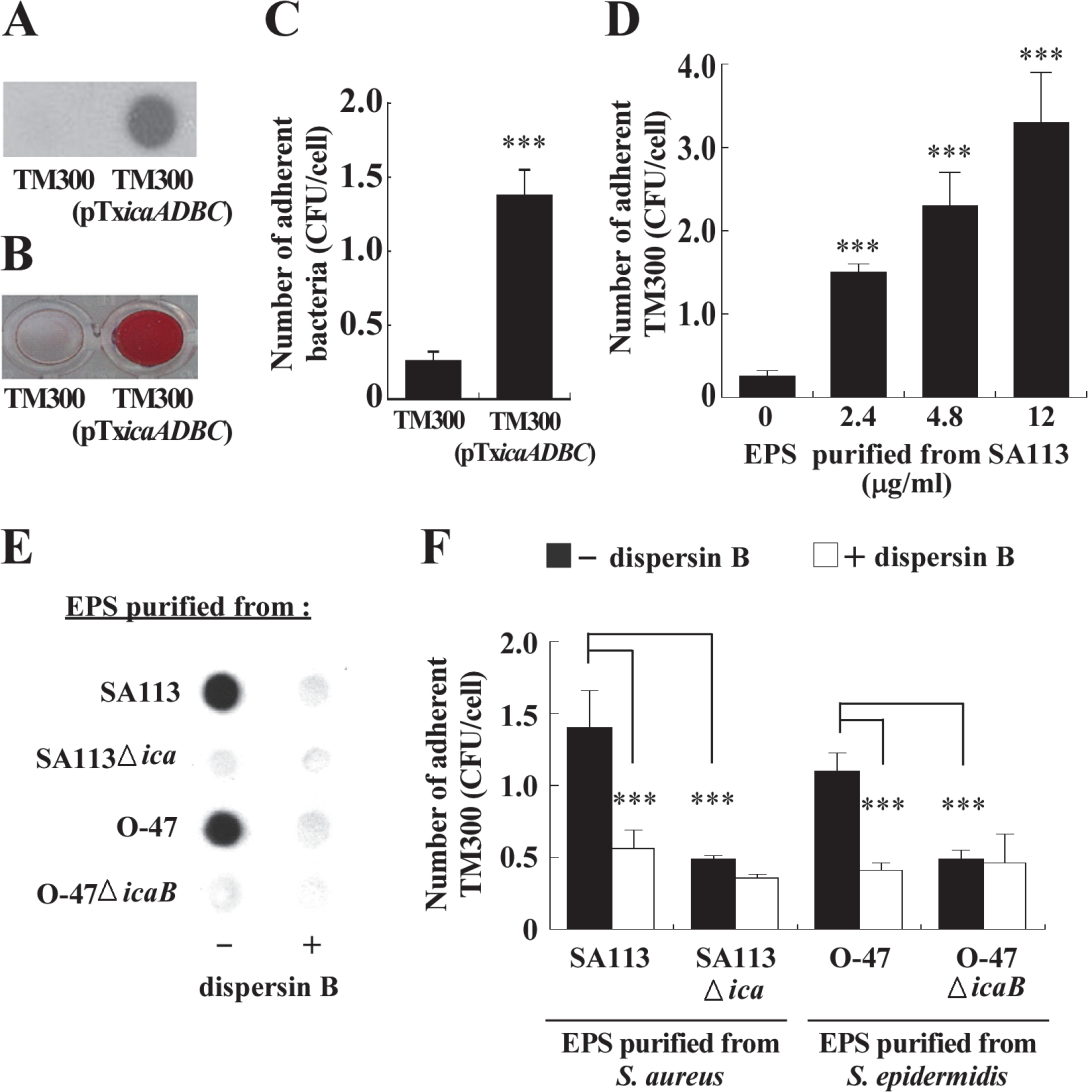
Effects of PIA/PNAG on the adherence of *S*. *carnosus* TM300 to epithelial cells. PIA/PNAG production (A), biofilm formation (B) and adherence of *S*. *carnosus* TM300 and *S*. *carnosus* TM300(pTX*icaADBC*) to RPMI 2650 cells (C) were determined. (D) EPS that was purified from *S*. *aureus* SA113 at various concentrations was mixed with *S*. *carnosus* TM300 and incubated with RPMI 2650 cells. The average number of *S*. *carnosus* TM300 that adhered to each RPMI2650 cell was determined. EPS (2.4 μg/ml) from *S*. *aureus* SA113, SA113Δ*ica*, *S*. *epidermidis* O-47 and *S*. *epidermidis* O-47Δ*icaB* was treated or untreated with 0.5 mg/ml dispersin B. The amounts of PIA/PNAG were determined by WGA-biotin (E). The average number of *S*. *carnosus* TM300 adhered to each RPMI2650 cell was determined by CFU enumeration (F). Significant differences are denoted with ***p-value < 0.001.

*S*. *carnosus* does not adhere to epithelial cells because it lacks substances that are required for cell adhesion [21]. This study investigates whether expressing PIA/PNAG or adding purified PIA/PNAG to *S*. *carnosus* TM300 enhances its adherence to epithelial cells. The results of this study confirmed that *S*. *carnosus* TM300 did not form PIA/PNAG and biofilms (Fig 4A and 4B). *S*. *carnosus* TM300 also had a low adherent ability to RPMI 2650 cells. The average number of adherent bacteria to each cell was 0.26 (Fig 4C). However, culturing *S*. *carnosus* TM300(pTX*icaADBC*) in a medium that contained xylose increased PIA/PNAG production (Fig 4A) and biofilm formation (Fig 4B). The presence of the PIA/PNAG-expressing plasmid also increased the number of bacteria that adhered to each RPMI 2650 cell by about fivefold to 1.36 (Fig 4C). Total exopolysaccharides (EPS), which include PIA/PNAG, were extracted from *S*. *aureus* SA113. The concentration of purified EPS was 60 μg/ml. Adding EPS at concentrations of 2.4, 4.8 and 12 μg/ml to *S*. *carnosus* TM300 increased the number of adhered bacteria to 1.5, 2.3 and 3.3 per cell, respectively (Fig 4D). Adding 2.4 μg/ml EPS that was purified from *S*. *epidermidis* O-47 also increased the number of *S*. *carnosus* TM300 adhered to each RPMI 2650 cell to 1.1 (Fig 4F). Pretreating 2.4 μg/ml EPS that had been purified from *S*. *aureus* SA113 or *S*. *epidermidis* O-47 with 0.5 mg/ml dispersin B, which specifically hydrolyses the glycosidic linkages in PIA/PNAG, reduced the numbers of adherent bacteria to 0.56 and 0.41 per cell, respectively (Fig 4F), revealing that adding PIA/PNAG increased the rates of adherence of *S*. *carnosus* TM300 to RPMI 2650 cells. The degradation of PIA/PNAG by dispersin B was also verified (Fig 4E). Meanwhile, EPS that was purified from *S*. *aureus* SA113Δ*ica* or *S*. *epidermidis* O-47Δ*icaB* increased the number of *S*. *carnosus* TM300 adhered to each RPMI 2650 cell to 0.48 (Fig 4F). However, treating the EPS that was purified from *S*. *aureus* SA113Δ*ica* or *S*. *epidermidis* O-47Δ*icaB* with dispersin B had little effect on the ability of EPS to promote the cellular adherence of *S*. *carnosus* TM300 (Fig 4F), showing that the EPS that was purified from the two wild-type strains may have contained polysaccharides other than PIA/PNAG, which promoted the adherence of *S*. *carnosus* TM300 to the epithelial cells.

### Impact of PIA/PNAG on *S*. *aureus* lung infections in C57BL/6 mice

**Fig 5:**
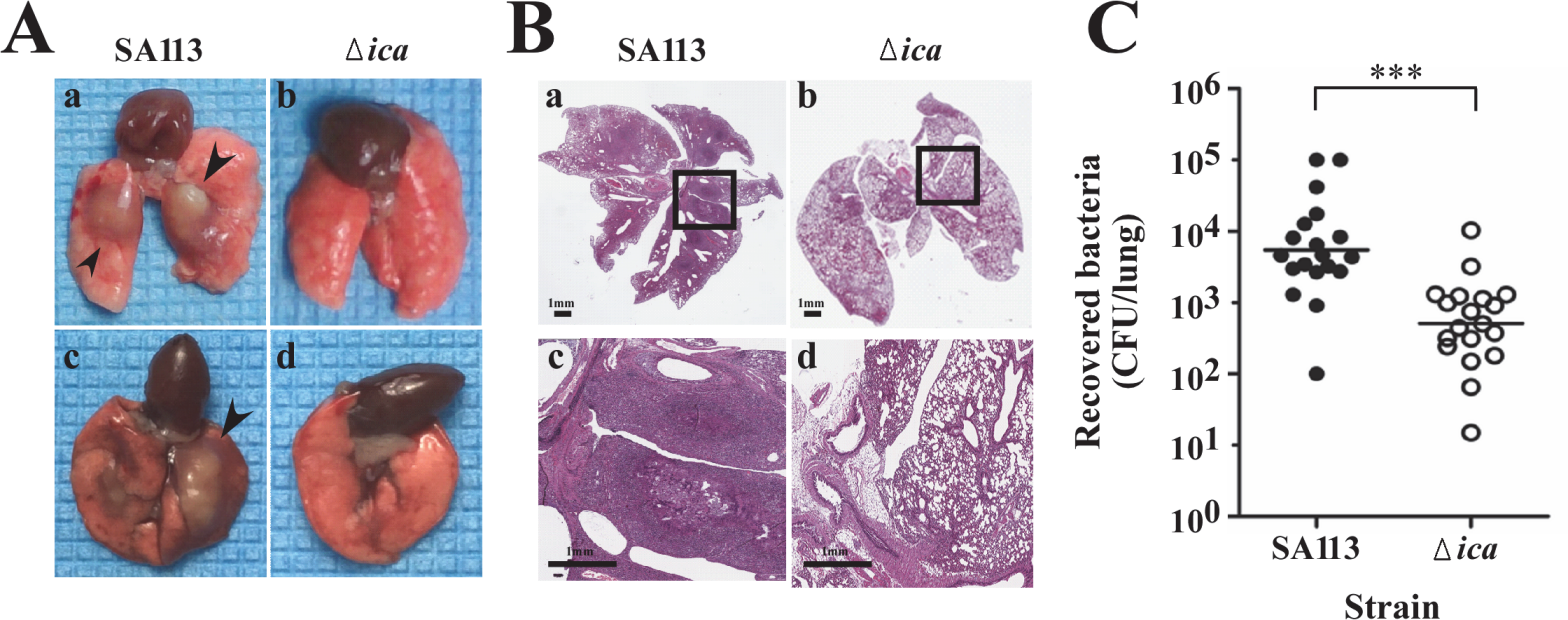
Effects of PIA/PNAG on lung infections in C57BL/6 mice. Mice were challenged with *S*. *aureus* SA113 or SA113Δ*ica* by intratracheal injection and were sacrificed three days after. (A) Lungs were removed from infected mice and photographed. Arrowheads indicated abscesses formed on SA113-infected lungs (a, c). (B) Lung tissues were fixed, embedded in paraffin, thin-sectioned and stained with H&E. The boxed areas in panels a and b are magnified in panels c and d. The images shown are representative of each group (n = 5 per group). (C) The number of bacteria recovered from the lung tissues of the infected mice was determined (n = 19 per group). The median values are indicated with horizontal lines. Significant differences are denoted with ***p-value < 0.001.

The effects of PIA/PNAG on cellular adhesion and infections *in vivo* were studied in a mouse model of lung infections. Mice were intratracheally injected with 1x10^9^ CFUs of *S*. *aureus* SA113 and SA113Δ*ica*. The lungs from the infected mice were photographed and examined histologically three days post-injection. Furthermore, the tissues were excised and homogenized to enumerate the bacterial loads in the infected lungs. As presented in Fig 5A, abscesses were observed in the lungs of mice that had been infected with *S*. *aureus* SA113 (Fig 5A-a, c). In contrast, none was observed in the lungs that were infected with SA113Δ*ica* (Fig 5A-b, d). Histological examination of the infected lungs revealed abscesses and inflammatory infiltration in the lungs that were infected with SA113 (Fig 5B-a, c) but not in those infected with SA113Δ*ica* (Fig 5B-b, d). Consistent with these histological findings, the bacterial burden that was detected in the lungs that had been infected with *S*. *aureus* SA113Δ*ica*, which had a mean of 5.1x10^2^ CFUs per lung, was less than that in the SA113-infected mice, which had a mean of 5.5x10^3^ CFUs per lung (Fig 5C), revealing that PIA/PNAG has a critical role in lung infections *in vivo*.

## Discussion

*S*. *aureus* is a leading cause of community-associated and nosocomial infections. This study demonstrates that a PIA/PNAG-deficient mutant strain, *S*. *aureus* SA113Δ*ica*, adheres less effectively to nasal epithelial cells than does the wild-type strain (Fig 1). Additionally, the adherence of the mutant to host cells is partially restored by transformation with pC*ica*, which carries the wild-type *ica* locus (Fig 1). The counting of 400 cells in the SEM images, verifies the involvement of PIA/PNAG in the attachment of *S*. *aureus* SA113 to RPMI 2650 cells (Fig 2). This study also reveals that both the external addition and endogenous expression of PIA/PNAG greatly increased the adherence of *S*. *carnosus* TM300 (Fig 4), indicating that PIA/PNAG synthesis promotes the adhesion of *S*. *carnosus* TM300 to epithelial cells. The results show that PIA/PNAG is crucial for staphylococcal adhesion to nasal epithelial cells.

Although PIA/PNAG is important to the adherence of *S*. *aureus* SA113 to epithelial cells, this study finds that a deletion of the *ica* operon does not completely abolish the cellular adherence by *S*. *aureus* (Fig 1). This is likely attributed to the synthesis of adhesins such as Eap, Emp, fibronectin-binding proteins (FnBPs), clumping factors (Clfs) and collagen-binding protein (Cna) [11,32–36], which are also responsible for the cellular adherence of *S*. *aureus*. Moreover, an earlier study demonstrated that, in addition to PIA, *S*. *epidermidis* expresses a 20-kDa polysaccharide, which promote bacterial adherence to surfaces and tissues [13]. *S*. *aureus* SA113Δ*ica* and *S*. *epidermidis* O-47Δ*icaB* may form similar exopolysaccharides that promote cellular adherence. The results explain why dispersin B-treated EPS from the two wild-type strains also increases the adherence of *S*. *carnosus* TM300 (Fig 4F). Furthermore, we found that treating purified EPS with proteinase K did not completely remove the proteins that were trapped in EPS. These bacterial surface proteins, in the absence of PIA/PNGA, may also promote the adherence of *S*. *carnosus* TM300 to cells. The results herein reveal that PIA/PNAG in EPS contributes to cellular adhesion of *S*. *carnosus* TM300.

Bacterial adherence to components of host tissues appears to be critical to their colonization and subsequent infection [10]. This study examines how PIA/PNAG affects bacterial adherence to tissues using a mouse model of lung infections and shows that the bacterial burden decreased significantly in the lungs infected with SA113Δ*ica* in comparison to those infected with the wild-type strain (Fig 5C). Additionally, microscopic and histopathologic analysis revealed that PIA/PNAG is associated with abscess formation and morphological changes of infected lung tissues (Fig 5A and 5B). These results indicate that PIA/PNAG of *S*. *aureus* is critical to invasive lung infection. According to a related study, the production of PIA/PNAG correlates with increased resistance to phagocytosis and killing by PMN [15]. The PIA/PNAG-mediated immune evasion and cellular adhesion may explain why PIA/PNAG-producing *S*. *aureus* establishes successful lung infections more efficiently than does the PIA/PNAG-deficient strain. Owing to the stability of plasmids in *S*. *aureus* following the infection of the mice and the choice not to use antibiotics in the infected animals, the animal studies did not include the complementary strain.

Staphylococcal adhesion to host components is vital to its colonization and infection. Related studies have described several host factors that serve as specific receptors for *S*. *aureus* binding [10]. Notably, extracellular matrix (ECM) components of host cells, including fibronectin (Fn), fibrinogen (Fg), vitronectin (Vn), elastin and collagen, are usually used as binding partners for the adhesion of *S*. *aureu*s to host cells [10]. However, PIA/PNAG is not involved in the ECM-mediated cellular adherence of *S*. *aureus* SA113 (S1 Fig). It is generally known that the deacetylation of PIA/PNAG by IcaB is essential for the production of positively charged PIA to stabilize its attachment to the cell surface [7]. Moreover, deacetylation of the PIA/PNAG polymer is essential for colonization, adhesion to epithelial cells, immune evasion and virulence in an animal infection model [14]. Therefore, electrostatic attraction between the anionic cell surfaces of epithelial cells and PIA/PNAG may contribute to the adherence effects of PIA/PNAG. This work reveals the importance of PIA/PNAG in *S*. *aureus* pathogenesis.

## Conclusions

In addition to adhering to host surfaces, pathogenic *S*. *aureus* must overcome host immune surveillance systems to sustain infection. This study demonstrates that PIA/PNAG contributes significantly to the adherence of *S*. *aureus* to nasal epithelial cells. Moreover, the results of the mouse model of lung infections suggest that PIA/PNAG plays a pivotal role in establishing successful *S*. *aureus* lung infections. Our findings indicate that PIA/PNAG is crucial to the pathogenesis of *S*. *aureus*.

## Supporting Information

S1 ARRIVE Checklist Completed ‘‘The ARRIVE Guidelines Checklist” for reporting animal data in this manuscript. (DOC)

S1 Fig Binding of bacteria to extracellular matrix proteins-coated plates. Bacteria were added to 96-well microtiter plates, which had been coated with 10 μg of fibronectin, fibrinogen, and collagen. Bacteria adhered to the wells were stained with 0.1% crystal violet, and measured at A_595_. *S*. *aureus* TM300 which does not bind to extracellular matrix proteins was used as a control.
(TIF)

## References

[1] Götz F. Staphylococci in colonization and disease: prospective targets for drugs and vaccines. Curr Opin Microbiol. 2004; 7: 477–487. 15451502 10.1016/j.mib.2004.08.014

[2] Wertheim HF, Melles DC, Vos MC, van Leeuwen W, van Belkum A, Verbrugh HA, et al The role of nasal carriage in *Staphylococcus aureus* infections. Lancet Infect Dis. 2005; 5: 751–762. 16310147 10.1016/S1473-3099(05)70295-4

[3] Wertheim HF, Vos MC, Ott A, van Belkum A, Voss A, Kluytmans JA, et al Risk and outcome of nosocomial *Staphylococcus aureus* bacteraemia in nasal carriers versus non-carriers. Lancet. 2004; 364: 703–705. 15325835 10.1016/S0140-6736(04)16897-9

[4] Götz F. Staphylococcus and biofilms. Mol Microbiol. 2002; 43: 1367–1378. 11952892 10.1046/j.1365-2958.2002.02827.x

[5] Gerke C, Kraft A, Sussmuth R, Schweitzer O, Götz F. Characterization of the N-acetylglucosaminyltransferase activity involved in the biosynthesis of the *Staphylococcus epidermidis* polysaccharide intercellular adhesin. J Biol Chem. 1998; 273: 18586–18593. 9660830 10.1074/jbc.273.29.18586

[6] Heilmann C, Schweitzer O, Gerke C, Vanittanakom N, Mack D, Götz F. Molecular basis of intercellular adhesion in the biofilm-forming *Staphylococcus epidermidis* . Mol Microbiol.1996; 20: 1083–1091. 8809760 10.1111/j.1365-2958.1996.tb02548.x

[7] Mack D, Fischer W, Krokotsch A, Leopold K, Hartmann R, Egge H, et al The intercellular adhesin involved in biofilm accumulation of *Staphylococcus epidermidis* is a linear beta-1,6-linked glucosaminoglycan: purification and structural analysis. J Bacteriol. 1996; 178: 175–183. 8550413 10.1128/jb.178.1.175-183.1996 PMC177636

[8] Krishna S, Miller LS. Host-pathogen interactions between the skin and *Staphylococcus aureus* . Curr Opin Microbiol. 2012; 15: 28–35. 10.1016/j.mib.2011.11.003 22137885 PMC3265682

[9] Clarke SR, Foster SJ. Surface adhesins of *Staphylococcus aureus* . Adv Microb Physiol. 2006; 51: 187–224. 17010697 10.1016/S0065-2911(06)51004-5

[10] Heilmann C. Adhesion mechanisms of staphylococci. Adv Exp Med Biol. 2011; 715: 105–123. 10.1007/978-94-007-0940-9_7 21557060

[11] Chavakis T, Wiechmann K, Preissner KT, Herrmann M. *Staphylococcus aureus* interactions with the endothelium: the role of bacterial "secretable expanded repertoire adhesive molecules" (SERAM) in disturbing host defense systems. Thromb Haemost. 2005; 94: 278–285. 16113816 10.1160/TH05-05-0306

[12] Xia G, Kohler T, Peschel A. The wall teichoic acid and lipoteichoic acid polymers of *Staphylococcus aureus* . Int J Med Microbiol. 2010; 300: 148–154. 10.1016/j.ijmm.2009.10.001 19896895

[13] Krevvata MI, Spiliopoulou A, Anastassiou ED, Karamanos N, Kolonitsiou F. Adherence of *Staphylococcus epidermidis* to human endothelial cells is associated with a polysaccharidic component of its extracellular mucous layer. Connect Tissue Res. 2011; 52: 183–189. 10.3109/03008207.2010.505309 20887232

[14] Vuong C, Kocianova S, Voyich JM, Yao Y, Fischer ER, DeLeo FR, et al A crucial role for exopolysaccharide modification in bacterial biofilm formation, immune evasion, and virulence. J Biol Chem. 2004; 279: 54881–54886. 15501828 10.1074/jbc.M411374200

[15] Vuong C, Voyich JM, Fischer ER, Braughton KR, Whitney AR, DeLeo FR, et al Polysaccharide intercellular adhesin (PIA) protects *Staphylococcus epidermidis* against major components of the human innate immune system. Cell Microbiol. 2004; 6: 269–275. 14764110 10.1046/j.1462-5822.2004.00367.x

[16] Costa AR, Henriques M, Oliveira R, Azeredo J. The role of polysaccharide intercellular adhesin (PIA) in *Staphylococcus epidermidis* adhesion to host tissues and subsequent antibiotic tolerance. Eur J Clin Microbiol Infect Dis. 2009; 28: 623–629. 10.1007/s10096-008-0684-2 19130107

[17] Iordanescu S, Surdeanu M. Two restriction and modification systems in *Staphylococcus aureus* NCTC8325. J Gen Microbiol. 1967; 96: 277–281. 10.1099/00221287-96-2-277 136497

[18] Cramton SE, Gerke C, Schnell NF, Nichols WW, Götz F. The intercellular adhesion (*ica*) locus is present in *Staphylococcus aureus* and is required for biofilm formation. Infect Immun. 1999; 67: 5427–5433. 10496925 10.1128/iai.67.10.5427-5433.1999 PMC96900

[19] Heilmann C, Gerke C, Perdreau-Remington F, Götz F. Characterization of Tn917 insertion mutants of *Staphylococcus epidermidis* affected in biofilm formation. Infect Immun. 1996; 64: 277–282. 8557351 10.1128/iai.64.1.277-282.1996 PMC173756

[20] Götz F. *Staphylococcus carnosus*: a new host organism for gene cloning and protein production. Soc Appl Bacteriol Symp Ser. 1990; 19: 49S–53S. 2119065 10.1111/j.1365-2672.1990.tb01797.x

[21] Rosenstein R, Nerz C, Biswas L, Resch A, Raddatz G, Schuster SC, et al Genome analysis of the meat starter culture bacterium *Staphylococcus carnosus* TM300. Appl Environ Microbiol. 2009; 75: 811–822. 10.1128/AEM.01982-08 19060169 PMC2632126

[22] Hanahan D, Jessee J, Bloom FR (1991) Plasmid transformation of *Escherichia coli* and other bacteria. Methods Enzymol. 1991; 204: 63–113. 1943786 10.1016/0076-6879(91)04006-a

[23] Waldron DE, Lindsay JA. Sau1: a novel lineage-specific type I restriction-modification system that blocks horizontal gene transfer into *Staphylococcus aureus* and between *S*. *aureus* isolates of different lineages. J Bacteriol. 2006; 188: 5578–5585. 16855248 10.1128/JB.00418-06 PMC1540015

[24] Lin TP, Chen CL, Chang LK, Tschen JS, Liu ST. Functional and transcriptional analyses of a fengycin synthetase gene, *fenC*, from *Bacillus subtilis* . J Bacteriol. 1999; 181: 5060–5067. 10438779 10.1128/jb.181.16.5060-5067.1999 PMC93996

[25] Lin MH, Shu JC, Huang HY, Cheng YC. Involvement of iron in biofilm formation by *Staphylococcus aureus* . PLoS One. 2012; 7: e34388 10.1371/journal.pone.0034388 22479621 PMC3313993

[26] Cramton SE, Ulrich M, Götz F, Doring G. Anaerobic conditions induce expression of polysaccharide intercellular adhesin in *Staphylococcus aureus* and *Staphylococcus epidermidis* . Infect Immun. 2001; 69: 4079–4085. 11349079 10.1128/IAI.69.6.4079-4085.2001 PMC98472

[27] Jang JH, Hia HC, Ike M, Inoue C, Fujita M, Yoshida, T. Acid hydrolysis and quantitative determination of total hexosamines of an exopolysaccharide produced by Citrobacter sp. Biotechnol Lett. 2005; 27: 13–18. 15685413 10.1007/s10529-004-6305-y

[28] Kaplan JB, Ragunath C, Ramasubbu N, Fine DH. Detachment of *Actinobacillus actinomycetemcomitans* biofilm cells by an endogenous beta-hexosaminidase activity. J Bacteriol. 2003; 185: 4693–4698. 12896987 10.1128/JB.185.16.4693-4698.2003 PMC166467

[29] Lin MH, Chang FR, Hua MY, Wu YC, Liu ST. Inhibitory effects of 1,2,3,4,6-penta-*O*-galloyl-beta-D-glucopyranose on biofilm formation by *Staphylococcus aureus* . Antimicrob Agents Chemother. 2011; 55: 1021–1027. 10.1128/AAC.00843-10 21173176 PMC3067108

[30] DuPage M, Dooley AL, Jacks T. Conditional mouse lung cancer models using adenoviral or lentiviral delivery of Cre recombinase. Nat Protoc. 2009; 4: 1064–1072. 10.1038/nprot.2009.95 19561589 PMC2757265

[31] Mack D, Siemssen N, Laufs R. Parallel induction by glucose of adherence and a polysaccharide antigen specific for plastic-adherent *Staphylococcus epidermidis*: evidence for functional relation to intercellular adhesion. Infect Immun. 1992; 60: 2048–2057. 1314224 10.1128/iai.60.5.2048-2057.1992 PMC257114

[32] Hussain M, Haggar A, Heilmann C, Peters G, Flock JI, Herrmann M. Insertional inactivation of Eap in *Staphylococcus aureus* strain Newman confers reduced staphylococcal binding to fibroblasts. Infect Immun. 2002; 70: 2933–2940. 12010982 10.1128/IAI.70.6.2933-2940.2002 PMC128007

[33] Hussain M, Becker K, von Eiff C, Schrenzel J, Peters G, Herrmann M. Identification and characterization of a novel 38.5-kilodalton cell surface protein of *Staphylococcus aureus* with extended-spectrum binding activity for extracellular matrix and plasma proteins. J Bacteriol. 2001; 183: 6778–6786. 11698365 10.1128/JB.183.23.6778-6786.2001 PMC95517

[34] Patti JM, House-Pompeo K, Boles JO, Garza N, Gurusiddappa S, Hook M. Critical residues in the ligand-binding site of the *Staphylococcus aureus* collagen-binding adhesin (MSCRAMM). J Biol Chem. 1995; 270: 12005–12011. 7744851 10.1074/jbc.270.20.12005

[35] Wertheim HF, Walsh E, Choudhurry R, Melles DC, Boelens HA, Miajlovic H, et al Key role for clumping factor B in *Staphylococcus aureus* nasal colonization of humans. PLoS Med. 2008; 5: e17 10.1371/journal.pmed.0050017 18198942 PMC2194749

[36] Sinha B, Francois PP, Nusse O, Foti M, Hartford OM, Vaudaux P, et al Fibronectin-binding protein acts as *Staphylococcus aureus* invasin via fibronectin bridging to integrin alpha5beta1. Cell Microbiol. 1999; 1: 101–117. 11207545 10.1046/j.1462-5822.1999.00011.x

