# Supplementary material for: Elucidating the Crucial Role of Poly N-Acetylglucosamine from Staphylococcus aureus in Cellular Adhesion and Pathogenesis
Source: PLoS One. 2015 Apr 15;10(4):e0124216. doi: 10.1371/journal.pone.0124216 (PMC4398431; doi:10.1371/journal.pone.0124216)

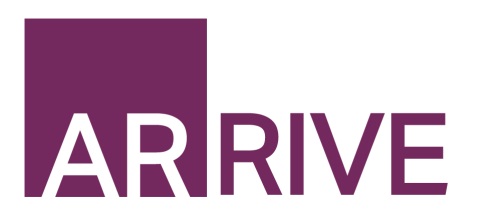


The ARRIVE Guidelines Checklist

Animal Research: Reporting In Vivo Experiments

Carol Kilkenny^1^, William J Browne^2^, Innes C Cuthill^3^, Michael Emerson^4^ and Douglas G Altman^5^

*^1^The National Centre for the Replacement, Refinement and Reduction of Animals in Research, London, UK, ^2^School of Veterinary Science, University of Bristol, Bristol, UK, ^3^School of Biological Sciences, University of Bristol, Bristol, UK, ^4^National Heart and Lung Institute, Imperial College London, UK, ^5^Centre for Statistics in Medicine, University of Oxford, Oxford, UK.*

|  | ITEM | RECOMMENDATION | Section/ Paragraph |
| --- | --- | --- | --- |
| 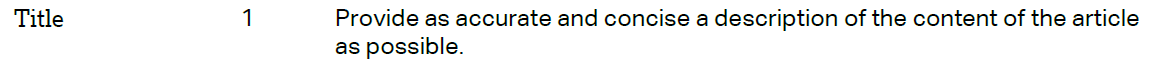 | | | Title page |
| 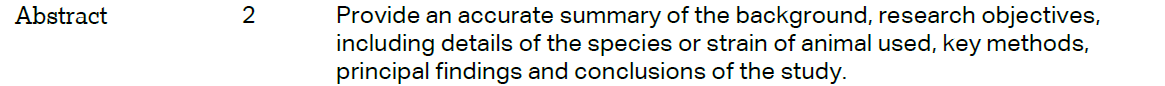 | | | Abstract |
| INTRODUCTION | | |  |
| 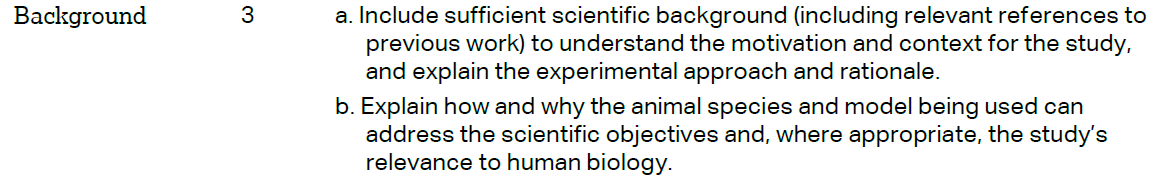 | | | Introduction  Paragraph 1-3  Ref. 14, 30 |
| 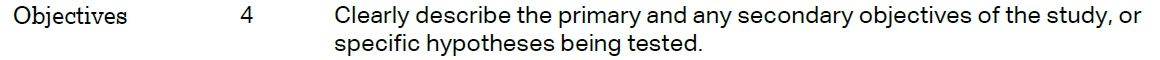 | | | Introduction  Paragraph 3 |
| METHODS | | |  |
| 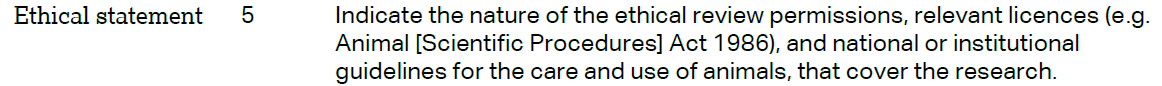 | | | Methods, (Mouse model of lung infections) |
| 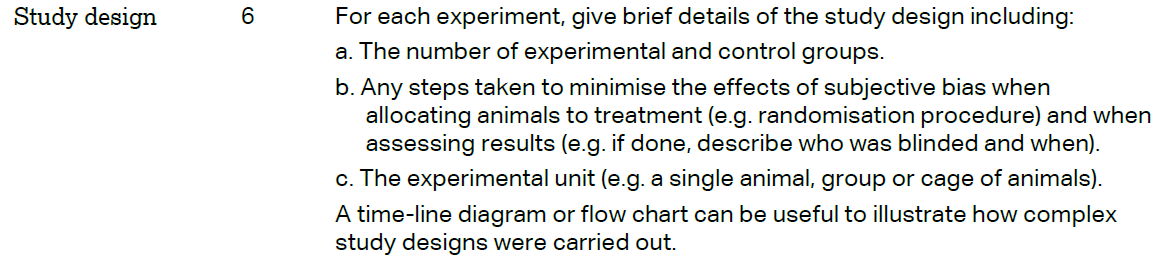 | | | Methods, (Mouse model of lung infections)  Results, section 3.  Figure 5 legend. |
| 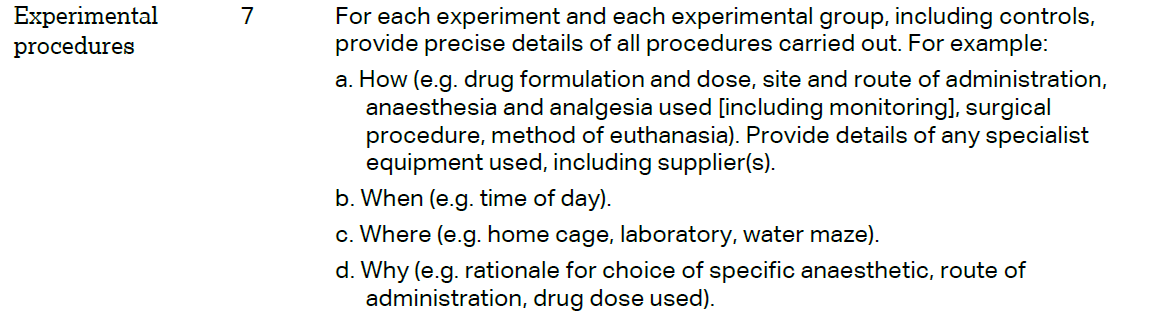 | | | Methods, (Mouse model of lung infections)  Results, section 3.  Figure 5 legend |
| 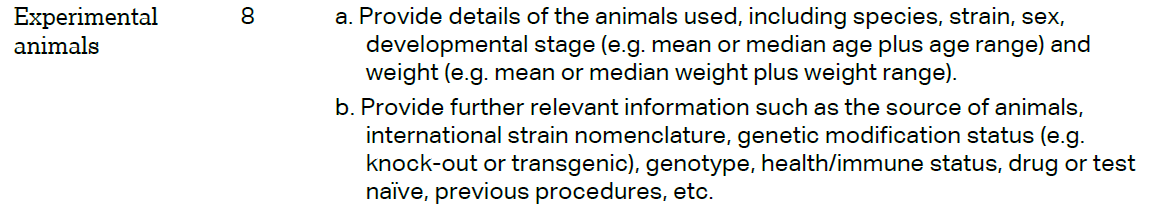 | | | Methods, (Mouse model of lung infections) |

The ARRIVE guidelines. Originally published in *PLoS Biology*, June 2010^1^

| 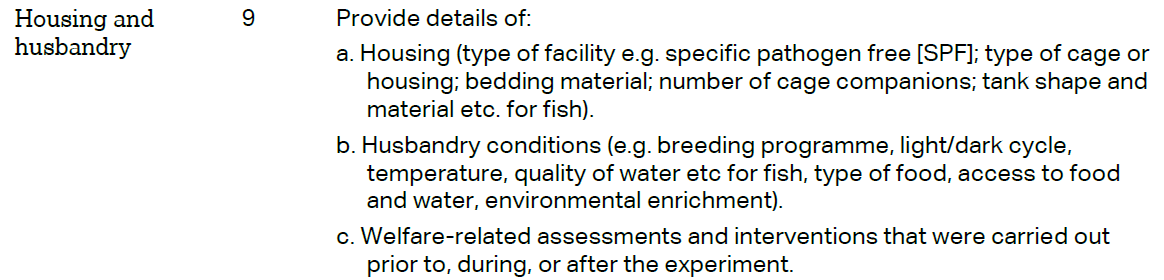 | Methods, (Mouse model of lung infections) | |
| --- | --- | --- |
| 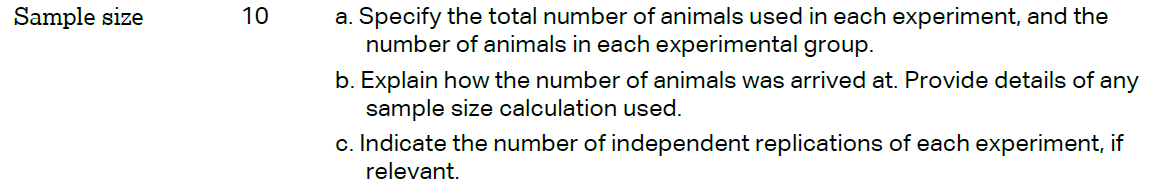 | Figure 5 legend  Methods, (Mouse model of lung infections) | |
| 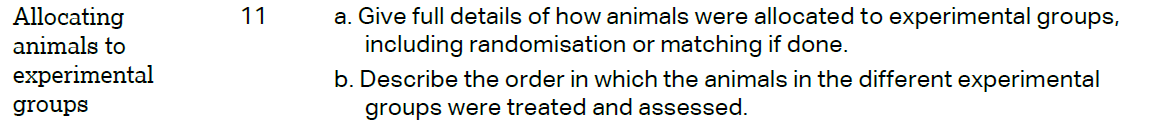 | Methods, (Mouse model of lung infections)  Result, Section 3  Figure 5 legend | |
| 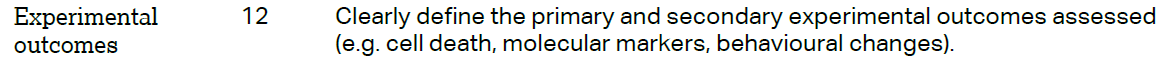 | Result, Section 3, | |
| 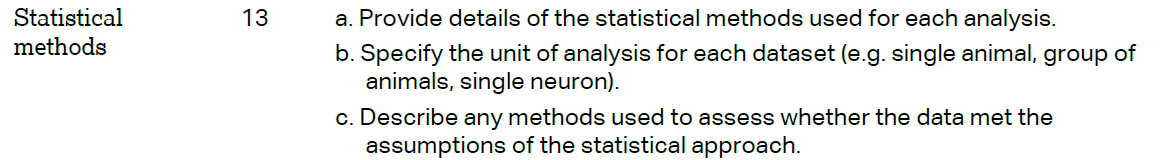 | Methods (Statistical analysis)  Figure 5 legend | |
| RESULTS |  | |
| 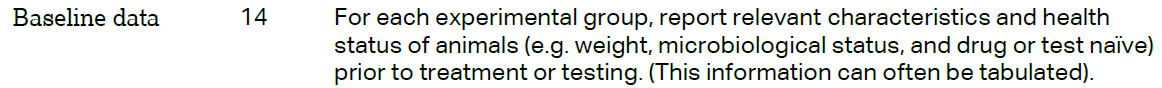 | Methods, (Mouse model of lung infections) | |
| 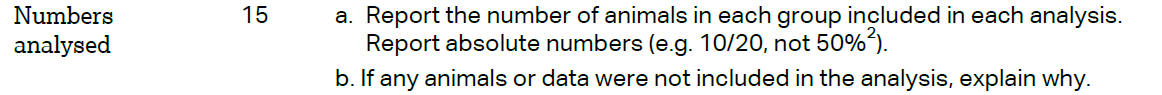 | Figure 5 legend | |
| 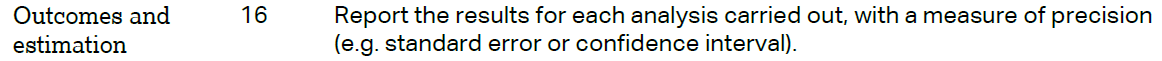 | Result, Section 3, Figure 5 legend | |
| 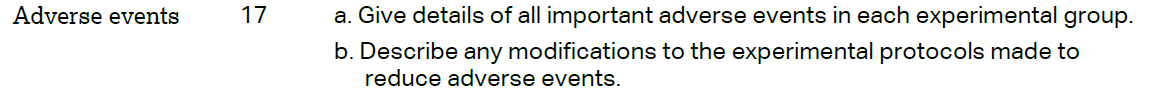 | Not applicable | |
| DISCUSSION |  | |
| 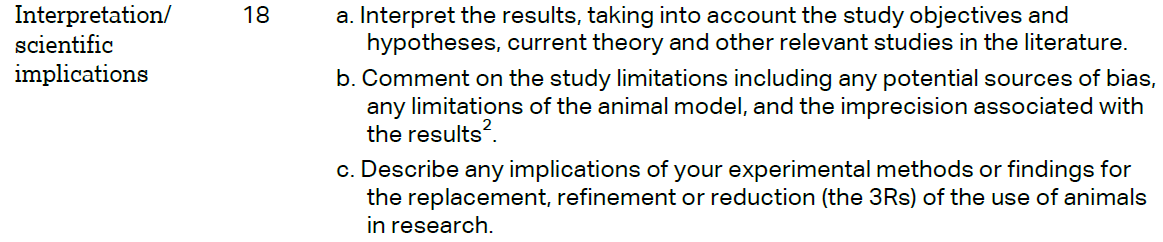 | Discussion  Paragraph 1-4 | |
| 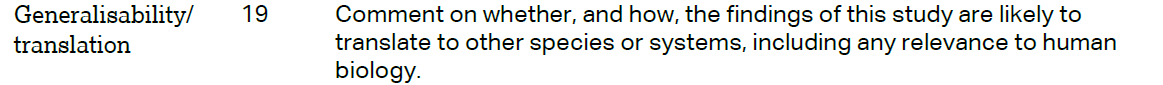 | Discussion  Paragraph 3 | |
| 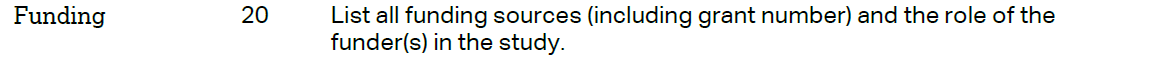 | Title page |  |


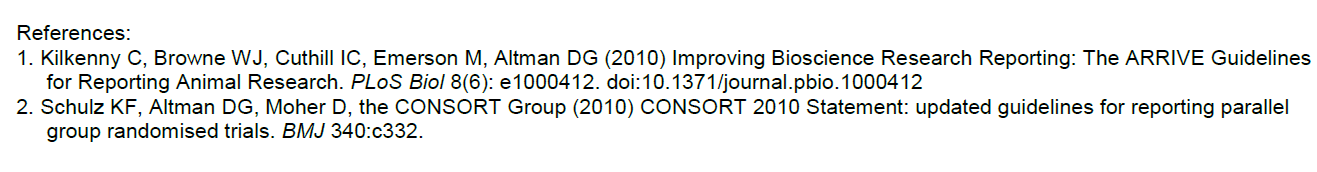

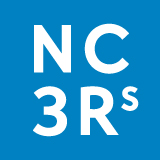

Supplement: S1 ARRIVE Checklist — (DOC) [file pone.0124216.s001.doc]
