# Supplementary figures and images for: Elucidating the Crucial Role of Poly N-Acetylglucosamine from Staphylococcus aureus in Cellular Adhesion and Pathogenesis
Source: PLoS One. 2015 Apr 15;10(4):e0124216. doi: 10.1371/journal.pone.0124216 (PMC4398431; doi:10.1371/journal.pone.0124216)

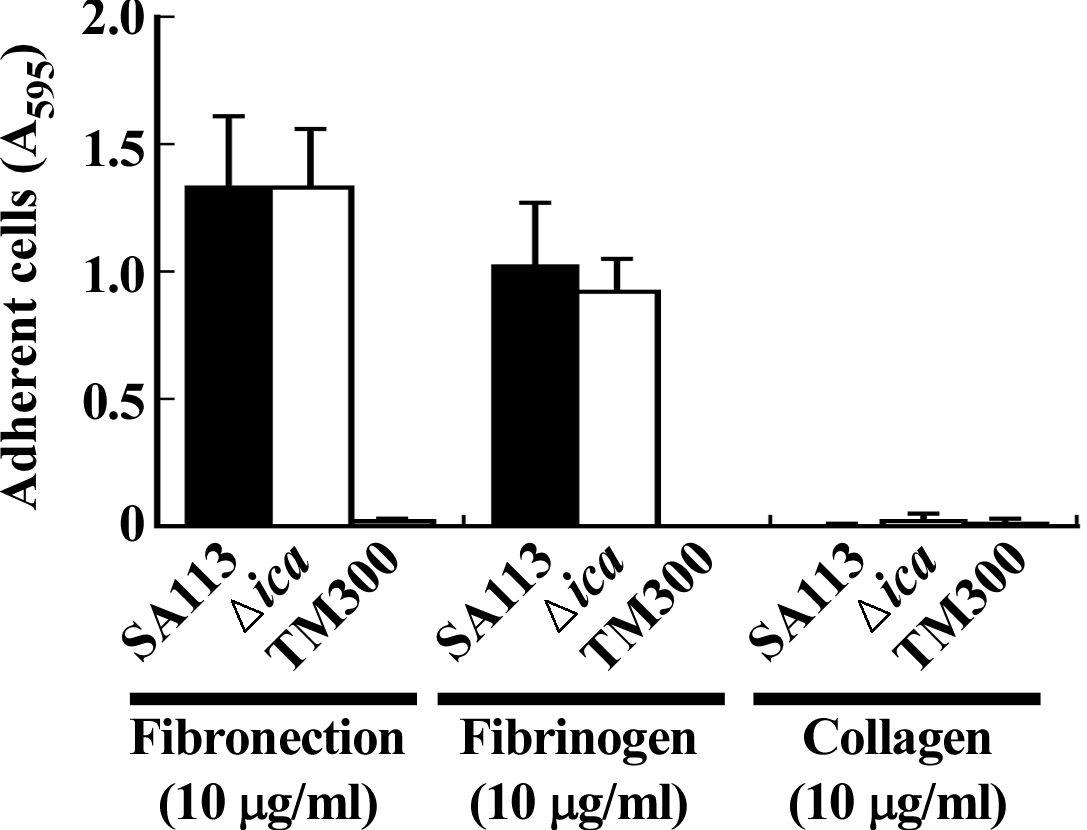

Supplement: S1 Fig — Bacteria were added to 96-well microtiter plates, which had been coated with 10 μg of fibronectin, fibrinogen, and collagen. Bacteria adhered to the wells were stained with 0.1% crystal violet, and measured at A595. S. aureus TM300 which does not bind to extracellular matrix proteins was used as a control. (TIF) [file pone.0124216.s002.tif]
